# Supplementary material for: Regulation of REM and Non-REM Sleep by Periaqueductal GABAergic Neurons
Source: Nat Commun. 2018 Jan 24;9:354. doi: 10.1038/s41467-017-02765-w (PMC5783937; doi:10.1038/s41467-017-02765-w)
Supplement: Supplementary file 1 — Supplementary Information [file 41467_2017_2765_MOESM1_ESM.pdf]

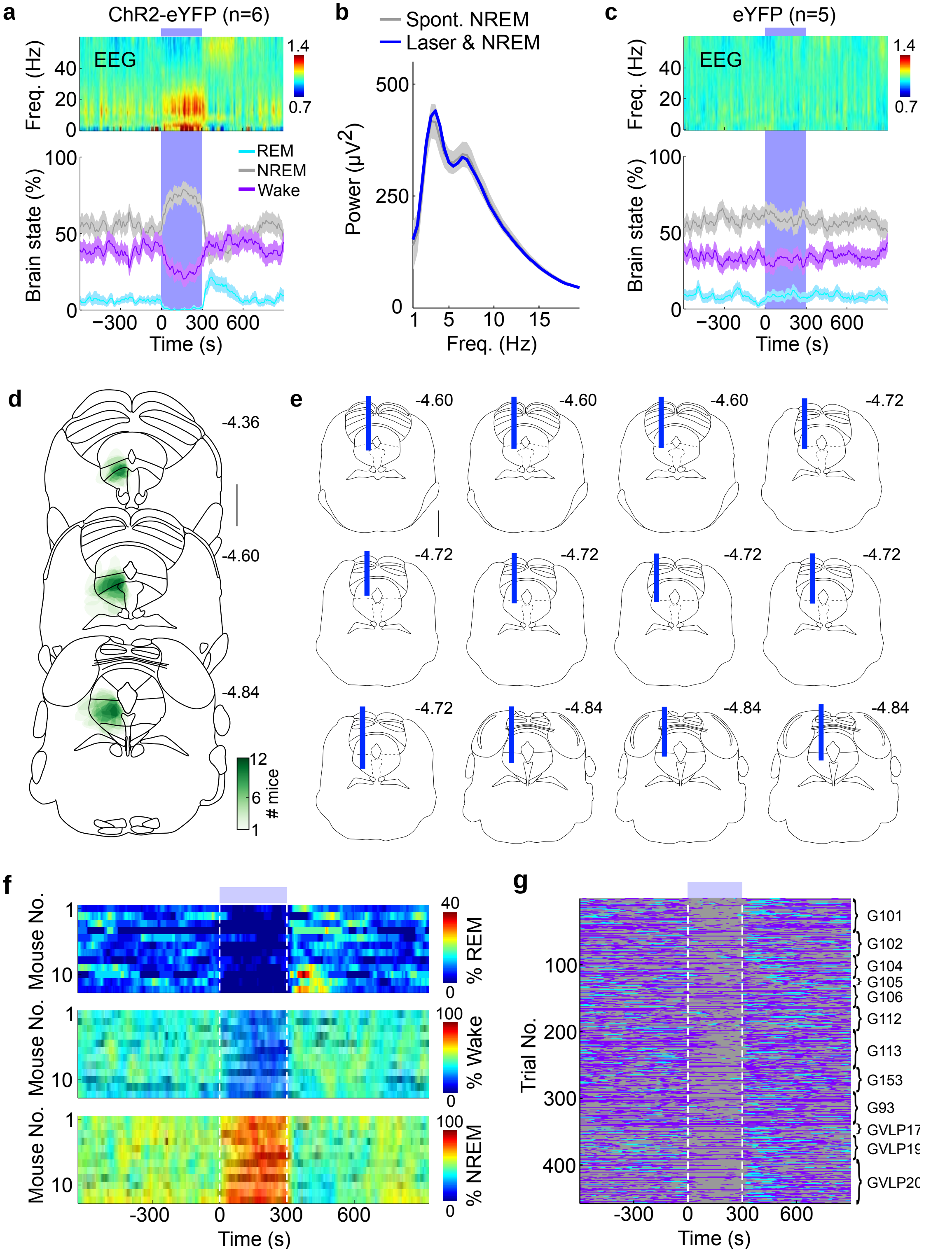

Supplementary Figure 1 Effects of laser stimulation in vlPAG of *GAD2-Cre* mice expressing ChR2-eYFP or eYFP alone and expression of ChR2 throughout vlPAG. (**a**) Effect of ChR2-mediated activation of GABAergic vlPAG neurons in additional mice (*n* = 6) that were not included in our previous study[1]. Top, average EEG spectrogram (normalized by the mean power in each frequency band). Bottom, percentage of REM,

NREM or wake states before, during, and after laser stimulation. The changes in REM, NREM, and wake induced by laser stimulation were highly significant ($P < 0.0001$, bootstrap). Shading, 95% confidence intervals (CI). Blue stripe, laser stimulation period (20 Hz, 300 s). (**b**) Comparison of EEG power spectra during spontaneous NREM sleep outside of laser stimulation periods (grey) and NREM sleep overlapping with laser stimulation (blue), averaged across all ChR2-eYFP mice (n = 12). Gray shading, ±s.e.m. for spontaneous NREM sleep. (**c**) Effect of laser stimulation on brain states in eYFP control mice (n = 5). Laser stimulation did not significantly change the percentage of any brain state ($P > 0.33$, bootstrap). (**d**) Expression of ChR2-eYFP in *GAD2-Cre* mice. For each mouse (n = 12), we determined the spread of ChR2-eYFP in three consecutive brain sections (from -4.36 mm to -4.84 mm along the rostrocaudal axis, where most of the virus expression was observed). The green color code indicates in how many mice the virus expression overlapped at the corresponding location. Scale bar, 1 mm. (**e**) Location of fiber tracts for optogenetic stimulation experiments. Each coronal section depicts the location of the optic fiber (blue bar) in each mouse (n = 12) used for optogenetic stimulation of vlPAG GABAergic neurons. Scale bar, 1 mm. (**f**) Percentage of REM (top), wake (middle), and NREM sleep (bottom) for each single mouse (n = 12). Each row corresponds to a single mouse and color-codes the brain state percentage averaged across all trials before, during, and after laser stimulation (300 s, 20 Hz). (**g**) All laser stimulation trials from 12 mice. Each row represents the color-coded brain state before, during, and after laser stimulation of a single trial. Each bracket on the right indicates all trials from a single mouse.

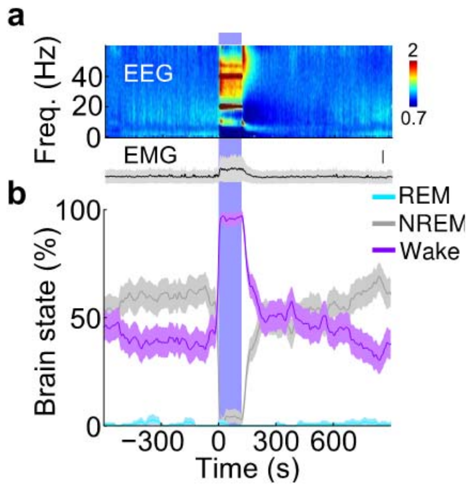

Supplementary Figure 2 Optogenetic activation of glutamatergic neurons in the vlPAG induces wakefulness. (**a**) Top, average EEG spectrogram (normalized by the mean power in each frequency band), averaged across all *VGLUT2-Cre* mice ($n = 4$). Bottom, average EMG amplitude. Scale bar 20 µV. Shading, ±s.e.m. (**b**) Bottom, percentage of REM, NREM, and wake states before, during, and after laser stimulation. Optogenetic activation of the glutamatergic neurons induced a strong increase in wakefulness ($P < 0.0001$, bootstrap), while suppressing NREM sleep ($P < 0.0001$). Blue stripe, laser stimulation period (20 Hz, 120 s).

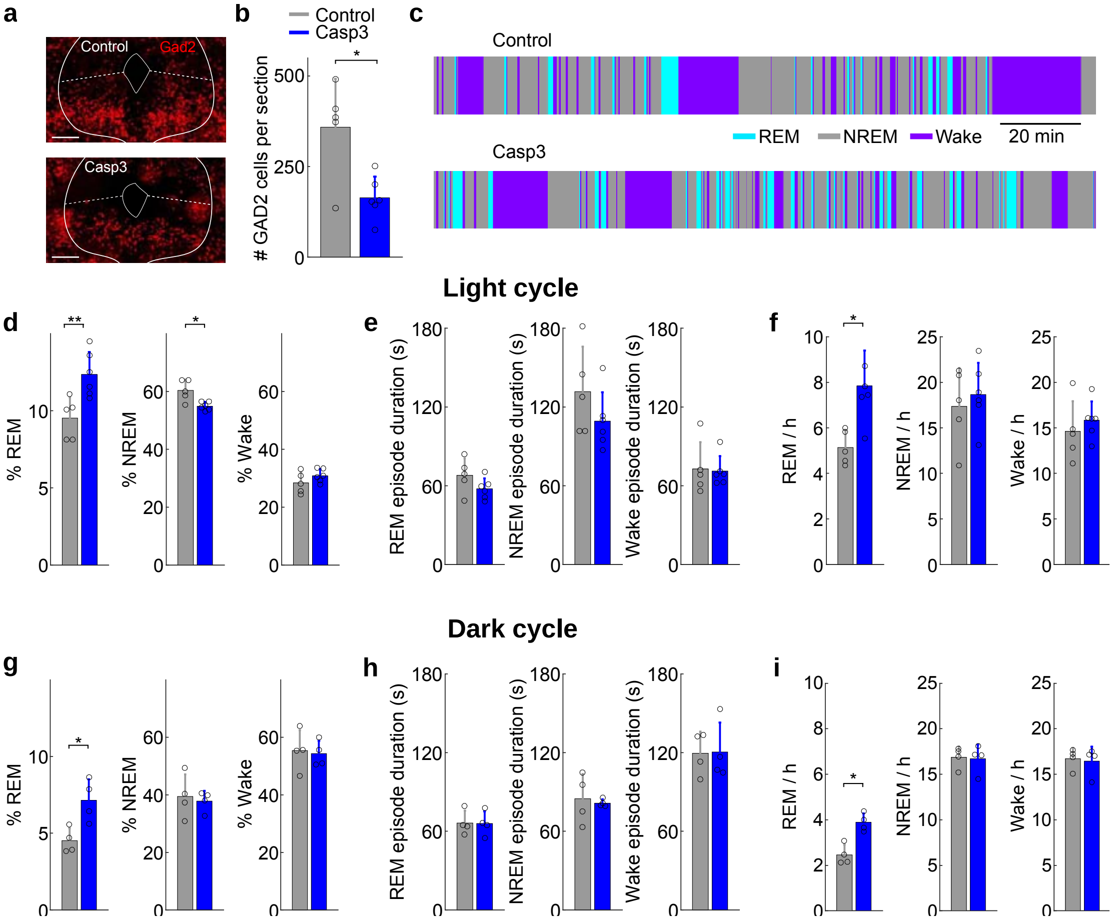

Supplementary Figure 3. Selective ablation of vlPAG GABAergic neurons increases REM sleep. (**a**) Top, fluorescence image from a control mouse showing the PAG. Red, FISH for mRNA encoding *GAD2* (scale bar, 250 μm). Bottom, fluorescence image from an experimental animal injected with AAV expressing pro-Caspase 3 (Casp3). Note the reduced number of *GAD2* neurons in the vlPAG compared to the control (top). (**b**) Number of *GAD2* cells per section (30 μm thick) in the vlPAG of control mice (n = 5) and mice expressing Caps3 (n = 6). On average, Casp3 expression reduced the number of GAD2 cells by 54.3% (P = 0.01, T(9) = -3.24; t-test). Each circle, data from one mouse. Error bar, ±s.d. (**c**) Top, 3 hr long hypnogram from a control mouse expressing eYFP recorded during the light cycle. Bottom, hypnogram recorded from a mouse expressing Casp3. (**d-f**) Percentage (**d**), average duration (**e**), and frequency (**f**) of REM, NREM, or wake states during the light cycle in control mice (n = 5, gray) and mice expressing Casp3 (n = 6, blue). In mice expressing Casp3, the percentage of REM sleep was significantly increased (P = 0.009, z = 50.0; Wilcoxon rank-sum test), due to an increased frequency of REM sleep episodes (P = 0.02, z = 49.0). In contrast, the amount of NREM sleep was significantly reduced (P = 0.02, z = 23.0). Each circle, data from one mouse. *P < 0.05, **P < 0.01, Wilcoxon rank-sum test. Error bar, ±s.d. (**g-i**) Percentage, average duration, and frequency of each brain state during the dark cycle in control mice (n = 4) and mice expressing Casp3 (n = 4). During the dark cycle, the percentage of REM sleep in mice expressing Casp3 was significantly increased (P = 0.03, z = 26.0), due to an increased frequency of REM sleep episodes (P = 0.03, z = 26.0).

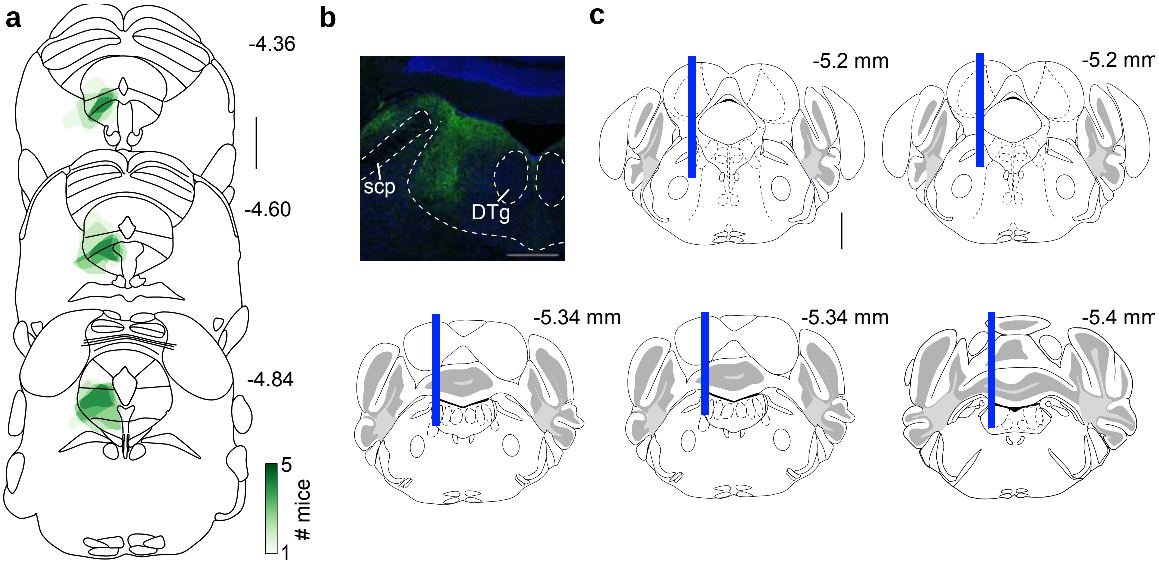

Supplementary Figure 4. Virus expression and optic fiber placement in axon fiber activation experiments. (**a**) Expression of ChR2-eYFP in *GAD2-Cre* mice at the injection site in vlPAG (n = 5). The virus expression was determined in three consecutive coronal sections. The color code indicates the number of mice, in which the virus expression overlapped at the corresponding location. Scale bar, 1 mm. (**b**) Expression of eYFP in the dorsolateral pons following AAV-eYFP injection into the vlPAG of a *GAD2-Cre* mouse. DTg, dorsal tegmental nucleus; scp, superior cerebellar peduncle. Scale bar, 500 µm. (**c**) Location of optic fibers in dorsolateral pons. The optic fibers were placed near the ventrolateral boundary of the pontine gray. Scale bar, 1 mm.

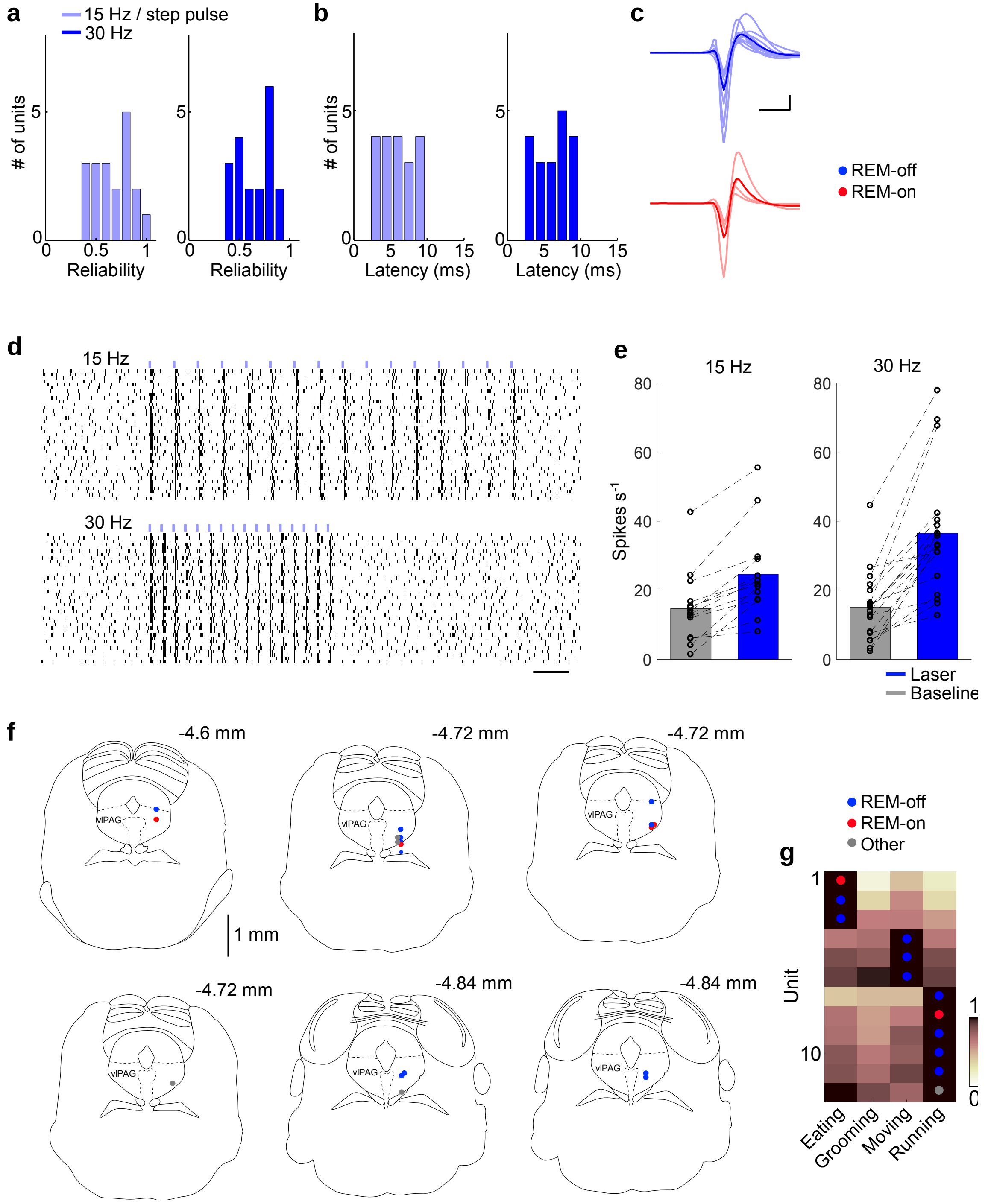

Supplementary Figure 5. Optogenetic identification of vlPAG GABAergic neurons. (**a**) Distribution of the reliability of laser-evoked spikes for the 19 identified GABAergic neurons, with 15 Hz or step pulse stimulation (left, 5/19 neurons were tested with step pulse and 14/19 with 15 Hz stimulation) or 30 Hz stimulation (right). The reliability was defined as the fraction of laser pulses followed by a spike. (**b**) Right, distribution of the latency of laser-evoked spikes for identified GABAergic neurons. Latency was defined as the timing of the first spike after the onset of each laser pulse. (**c**) Top, individual waveforms of GABAergic REM-off units (light blue) and average across units (dark

blue). Bottom, individual (light red) and average (dark red) waveforms of GABAergic REM-on units. Scale bars, 0.5 ms, 0.2 mV. (**d**) Raster plots for 15 and 30 Hz stimulation of an example unit with a relatively high baseline firing rate (24.7 spikes s^{-1}). Scale bar, 100 µs. (**e**) Firing rates during 15 or 30 Hz laser stimulation and baseline activity during the preceding interval (1 s for 15 Hz and 0.5 s for 30 Hz, matching the duration of laser stimulation in each case). Each line indicates the firing rates of a single unit. Units for which we presented a step pulse instead of 15 Hz stimulation are not shown on the left. Laser stimulation significantly increased the firing rates for both 15 Hz (n = 14 units, p = 0.0001, z = -3.3, Wilcoxon sign-rank test) and 30 Hz (n = 19 units, p = 0.0001, z = -3.8). Additionally, the firing rates of each single unit were significantly increased across stimulation trials for both conditions (15 Hz, $p < 0.04$ and 30 Hz, $p < 0.02$; Wilcoxon sign-rank test). (**f**) Positions of the 19 identified vlPAG GABAergic neurons from 6 mice. Each dot indicates one neuron. Blue, REM-off neurons; red, REM-on neurons; gray – other neurons. (**g**) Relative firing rates of vlPAG GABAergic neurons during different wakeful behaviors. Most vlPAG GABAergic neurons showed the highest wake activity during moving or running. Only neurons from recording sessions in which the animal was engaged in all four behaviors are shown (n = 12).

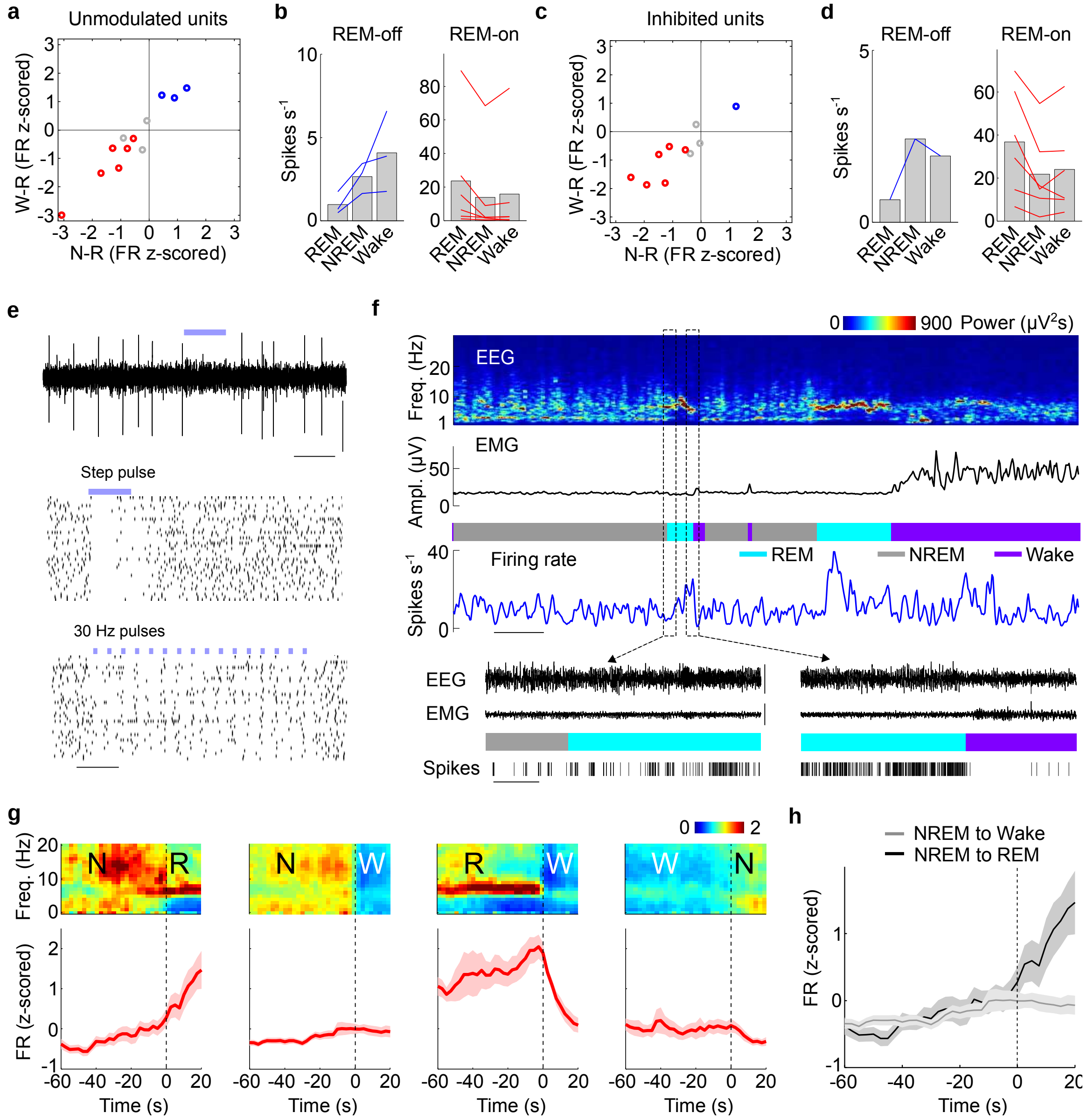

Supplementary Figure 6. Sleep-wake activity of unidentified vlPAG neurons. (**a**) Firing rates of laser-unmodulated units from 6 mice. Unmodulated units include all neurons that were not significantly modulated by laser stimulation, as quantified by SALT (see Methods). W, Wake; R, REM; N, NREM. Blue, significant REM-off neurons ($P < 0.05$, Wilcoxon rank-sum test, post-hoc Bonferroni correction); red, significant REM-on neurons; gray, other neurons. (**b**) Firing rates of laser-unmodulated REM-off (left) and REM-on (red) neurons during different brain states. Each line shows firing rates of one unit; gray bar, average across units. (**c**) Firing rate of laser-inhibited units. Inhibited units include neurons that were significantly modulated by laser stimulation, as quantified by SALT, and whose firing rate was significantly decreased during the 30 Hz laser stimulation period. (**d**) Firing rates of laser-inhibited REM-off (left) and REM-on (red) neurons during different brain states (n = 12). (**e**) Example of an inhibited vlPAG unit. Top, raw trace showing spikes before, during, and after laser stimulation. Blue bar, laser

step pulse (100 ms). Scale bars, 100 ms, 0.5 mV. Middle, spike raster showing multiple trials of laser stimulation with laser step pulse. Bottom, spike raster showing multiple trials of laser stimulation at 30 Hz. Scale bar, 100 ms. (**f**) Firing rate of an inhibited vlPAG neuron (blue) along with EEG spectrogram, EMG amplitude, and color-coded brain state (scale bar, 120 s). The timing of single spikes (vertical ticks) is depicted on an expanded time scale (indicated by black boxes) along with EEG, EMG raw traces (scale bars, 5 s, 0.5 mV). (**g**) Average EEG spectrogram (upper, normalized by the mean power in each frequency band) and mean firing rate (*z*-scored) of unidentified REM-on neurons (lower) at brain state transitions. The REM-on neurons include both laser-unmodulated (**a**, **b**) and laser-inhibited neurons (**c**, **d**). Shading, ±s.e.m. (**h**) The firing rates of REM-on neurons during NREM episodes preceding wake or REM episodes were not significantly different (n =12, P = 0.31, $T(11)$ = 1.06, paired t-test).

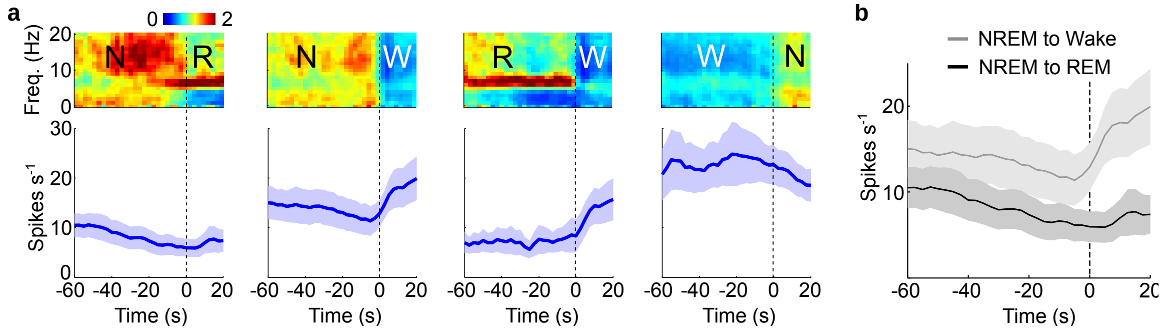

Supplementary Figure 7. Non-normalized firing rate of vlPAG GABAergic neurons during brain state transitions. (**a**) Average EEG spectrogram (upper, normalized by the mean power in each frequency band) and non-normalized mean firing rate of GABAergic REM-off neurons (lower) at brain state transitions (n =11). Shading, ±s.e.m. (**b**) The firing rates during NREM episodes preceding wake were significantly higher than those preceding REM episodes (P = 0.002, paired t-test).

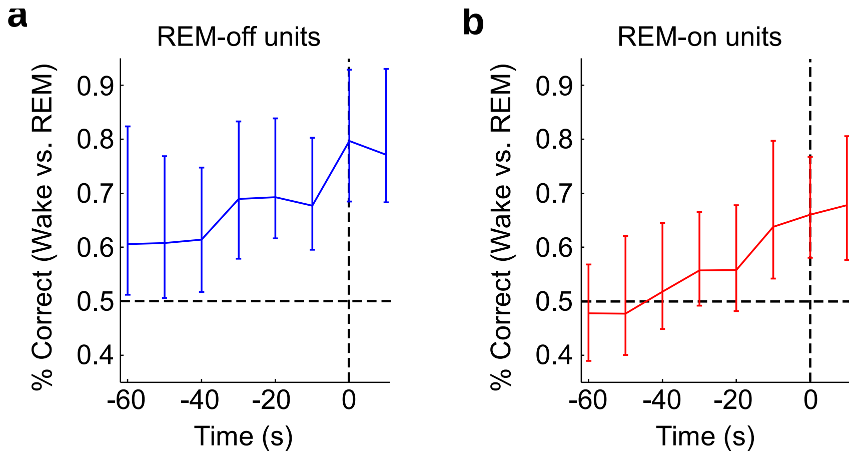

Supplementary Figure 8. Prediction of transition to REM or wake based on vlPAG NREM firing rates. (**a**) Linear discrimination analysis evaluating how well a NREM→REM or NREM→Wake transition can be predicted from the preceding NREM activity of single GABAergic REM-off neurons (n = 11). The prediction accuracy was above the chance level (horizontal dashed line) even 60 s before the transition (P = 0.04, bootstrap). Error bars, 95% CI. (**b**) Prediction of NREM→REM or NREM→Wake transitions based on the preceding NREM activity of single REM-on neurons including both laser-unmodulated and laser-inhibited neurons (n = 12, red units in Supplementary Fig. 6a-d). In contrast to REM-off neurons, the prediction accuracy was above the chance level only 10 s before the transition.

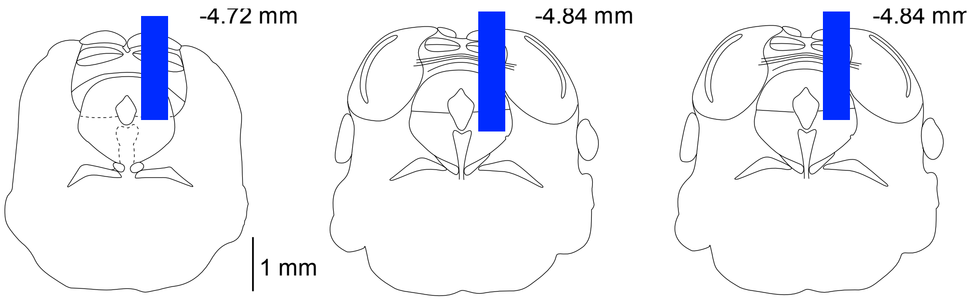

Supplementary Figure 9. Position of GRIN lenses within the vlPAG of *GAD2-Cre* mice used for imaging vlPAG GABAergic neurons. Each diagram depicts the section where the lesion caused by the GRIN lens was largest along the rostrocaudal axis. Blue bar, GRIN lens; scale bar, 1 mm.

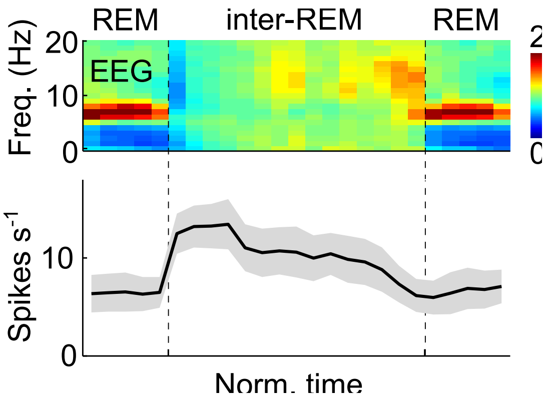

Supplementary Figure 10. Non-normalized firing rate of vlPAG GABAergic neurons during inter-REM periods. Shown are average normalized EEG spectrogram (upper) and non-normalized mean firing rate of significant REM-off vlPAG GABAergic neurons (lower) during two successive REM episodes and the inter-REM interval. To average across multiple inter-REM periods REM and inter-REM durations were time-normalized.

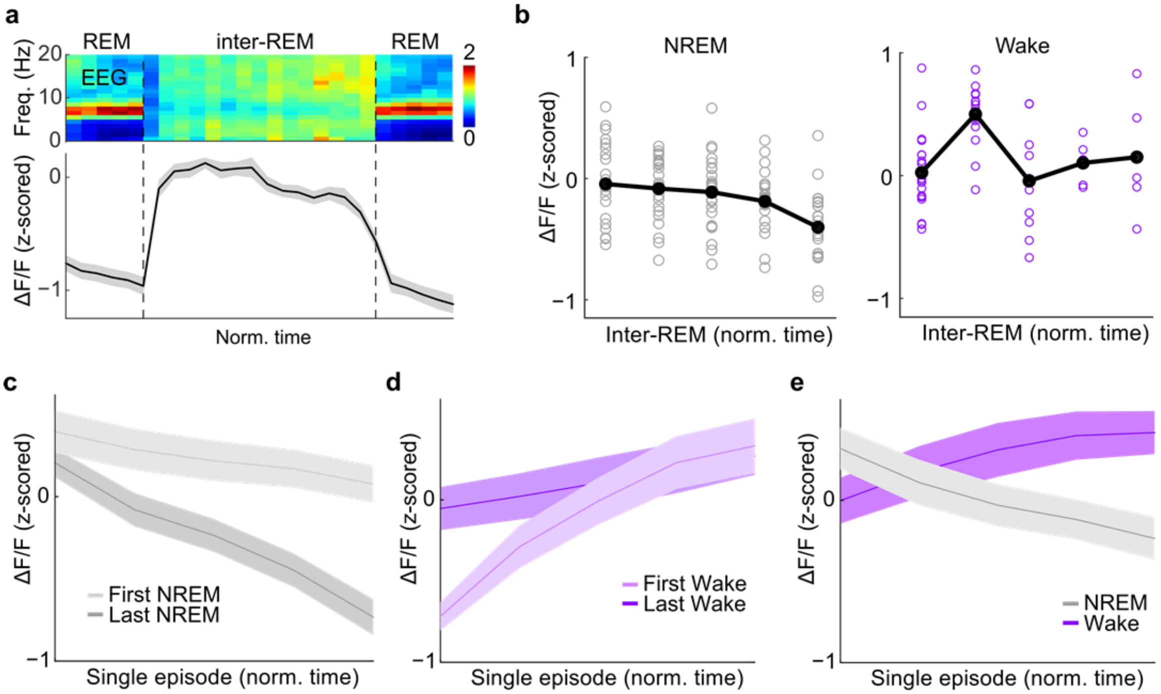

Supplementary Figure 11. Slow modulation of vlPAG GABAergic neuron activity measured with calcium imaging. (**a**) Average normalized EEG spectrogram (upper) and mean calcium activity (ΔF/F, *z*-scored) of significant REM-off ROIs (lower) during two successive REM episodes and the inter-REM interval. Each REM episode and inter-REM interval was temporally compressed to unit length before the calcium traces were averaged over multiple episodes/intervals and across ROIs (n = 23). Shading, ±s.e.m. (**b**) Calcium activity (ΔF/F, *z*-scored) during NREM (left) and wake (right) episodes within different segments of the inter-REM interval. Each inter-REM interval was divided into 5 equally sized bins, and NREM or wake activities were averaged for each bin. Each symbol represents the average NREM or wake activity of an ROI. The average NREM activity decreased during the inter-REM period (R = -0.36, P = 7.3 x 10^{-5}, $T(21)$ = -1.77, linear regression), while the wake activity showed no significant trend (R = 0.04, P = 0.76, $T(23)$ = -0.18). Black line, average calcium activity of each bin. (**c**) Mean calcium activity during the first (light gray) and last (dark gray) NREM episodes of each inter-REM interval. Each NREM episode was temporally compressed to unit duration before the *z*-scored calcium activity was averaged over episodes and across ROIs. The activity during the first NREM period was significantly higher than during the last period (n = 23 ROIs, P = 0.00015, $T(22)$ = 4.41, paired *t*-test). Shading, ±s.e.m. (**d**) Mean calcium activity during the first (light purple) and last (dark purple) wake episodes of each inter-REM interval. Each wake episode was temporally compressed to unit duration before averaging (P = 0.25, $T(22)$ = -1.17). (**e**) Mean calcium activity during all NREM (gray) and wake (purple) episodes. Note that the calcium activity decreased during NREM (R = -0.36, P = 2.9 x 10^{-24}, $T(21)$ = -1.77) but increased during wake episodes (R = 0.27, P = 1.7 x 10^{-13}, $T(21)$ = 1.29).

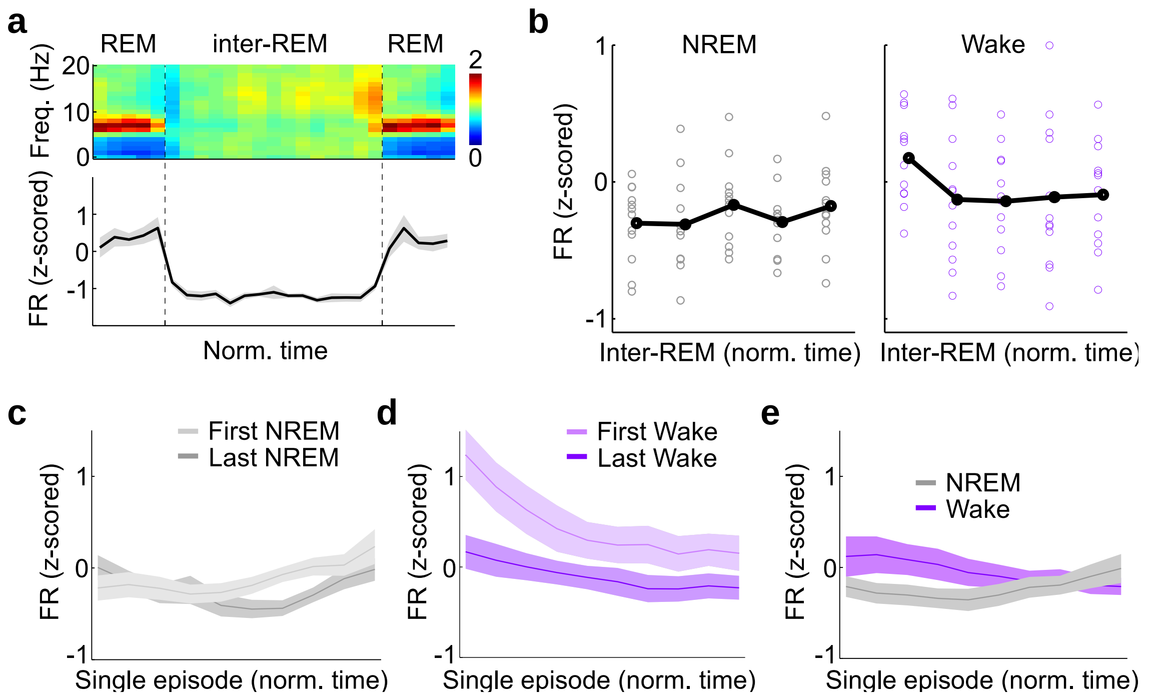

Supplementary Figure 12. Firing rates of vlPAG REM-on neurons during inter-REM interval. (**a**) Average normalized EEG spectrogram (upper) and mean firing rate (*z*-scored) of significant REM-on vlPAG neurons (lower) during two successive REM episodes and the inter-REM interval. The REM-on neurons include both laser-unmodulated and laser-inhibited neurons (n = 12, red units in Supplementary Fig. 6a-d). Each REM episode and inter-REM interval was temporally compressed to unit length before the firing rates were averaged over multiple episodes/intervals and across REM-on neurons. Shading, ±s.e.m. (**b**) Firing rate (FR, *z*-scored) during NREM (left) and wake (right) episodes within different segments of the inter-REM interval. Each inter-REM interval was divided into 5 equally sized bins, and NREM or wake firing rates were averaged for each bin. Each symbol represents the average NREM or wake firing rate of a unit. Neither the NREM nor wake activity showed a consistent change across the inter-REM period (NREM, R = 0.13, P = 0.33, $T(58)$ = 0.10; Wake, R = -0.17, P = 0.19, $T(58)$ = -1.32; linear regression). Black line, average firing rate of each bin. (**c**) Mean firing rates of vlPAG REM-on neurons during the first (light gray) and last (dark gray) NREM episodes of each inter-REM interval. Each NREM episode was temporally compressed to unit duration before the *z*-scored firing rate was averaged over episodes and across cells. The firing rates during the last and first NREM episode were not significantly different (P = 0.19, $T(11)$ = -1.39, paired *t*-test). Shading, ±s.e.m. (**d**) Mean firing rates of REM-on units during the first (light purple) and last (dark purple) wake episodes of each inter-REM interval. Each wake episode was temporally compressed to unit duration before averaging. The firing rate during the first wake period was significantly higher than during the last one (P = 0.04, $T(11)$ = 2.36, paired *t*-test). (**e**) Mean firing rates of REM-on units during all NREM (gray) and wake (purple) episodes. The firing rate showed no systematic change during each NREM episode (R = 0.18, P = 0.063, $T(108)$ = 1.88, linear regression) and a slight decrease during the wake episode (R = -0.24, P = 0.01, $T(108)$ = -2.61, linear regression).

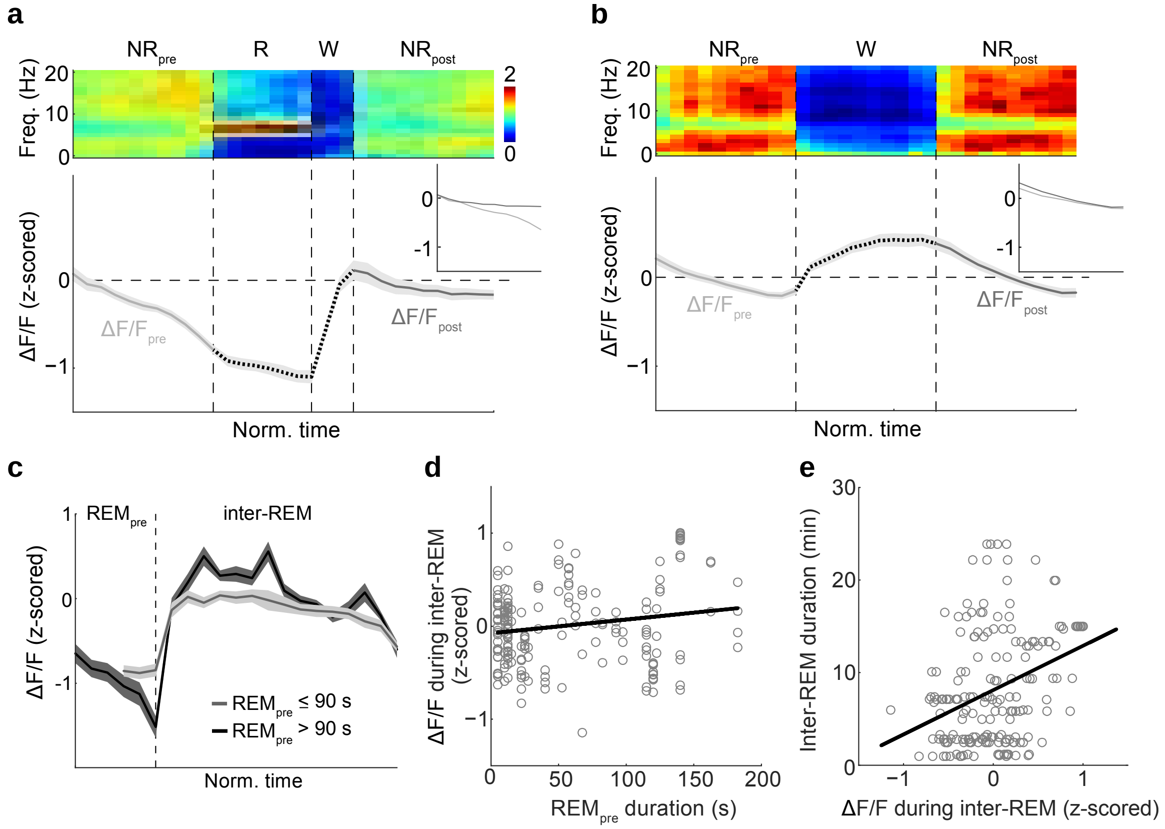

Supplementary Figure 13. Homeostatic modulation of vlPAG GABAergic neuron activity measured with calcium imaging. (**a**) Average normalized EEG spectrogram (upper) and mean calcium activity (ΔF/F, *z*-scored) of significant REM-off ROIs (lower, n = 23) during the NREM→REM→Wake→NREM transition sequence. Each REM, NREM, or wake episode was temporally compressed to unit length before the calcium activity was averaged over multiple sequences and across ROIs. Shading, ±s.e.m. Inset, the calcium activity during the NREM episodes preceding REM (NR_{pre}) was significantly higher than during NREM episodes following REM (NR_{post}; P = 0.025, $T(22)$ = -2.39, paired *t*-test). (**b**) Similar to (**a**), but for the NREM→Wake→NREM transition sequence. Note that without the intervening REM episode, the calcium activities were not significantly different during NR_{pre} and NR_{post} (P = 0.085, $T(22)$ = -1.81). (**c**) The calcium activity of REM-off ROIs during the inter-REM interval following a long (> 90 s) REM episode was higher than that following a short (≤ 90 s) REM episode (P = 0.028, $T(22)$ =-2.38, paired *t*-test). (**d**) Correlation between REM episode duration and calcium activity during the subsequent inter-REM interval. Each dot represents the activity of an ROI during a single inter-REM interval (n = 189). Line, linear fit (R = 0.18, P = 0.016, $T(187)$ = 2.50, linear regression). (**e**) Correlation between calcium activity during inter-REM interval and duration of the interval. Each dot represents the activity of an ROI during a single inter-REM interval (n = 189). Line, linear fit (R = 0.35$, $P = 1.2 \times 10^{-6}$, $T(187)$ = 5.11).

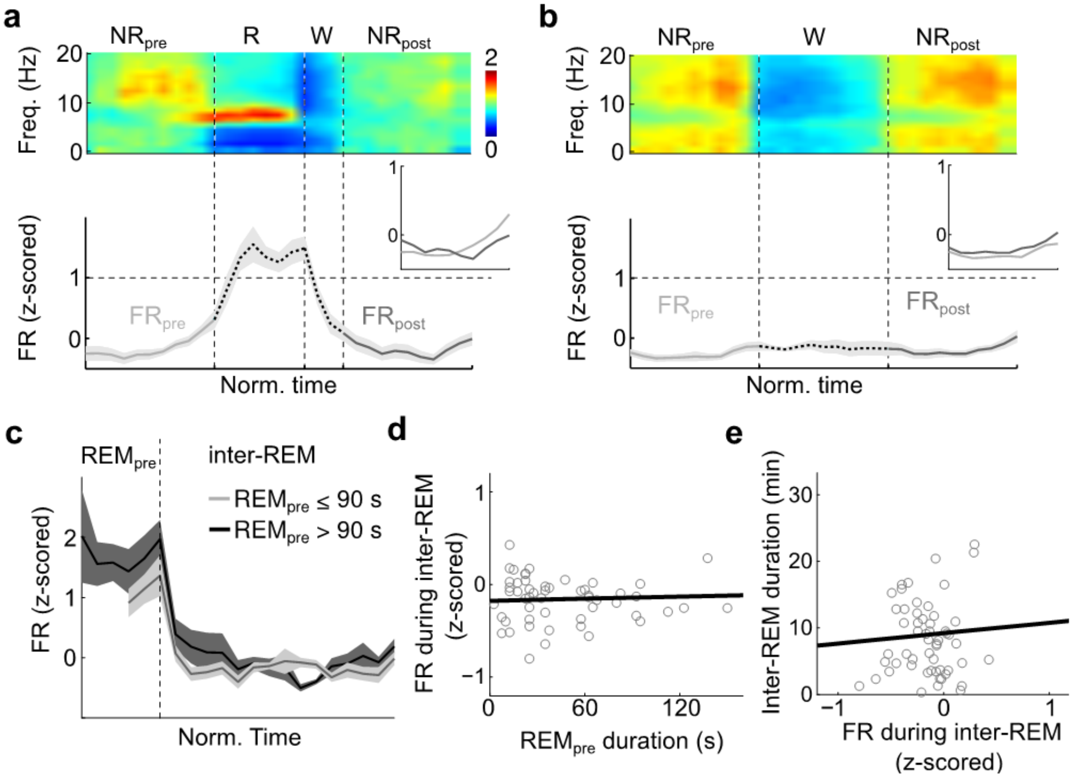

Supplementary Figure 14. Firing rates of REM-on neurons are not modulated by homeostatic REM sleep pressure. (**a**) Average normalized EEG spectrogram (upper) and mean firing rate (*z*-scored) of significant REM-on neurons (lower) during the NREM→REM→Wake→NREM transition sequence. REM-on neurons include both laser-unmodulated and laser-inhibited units (n = 12). Each REM, NREM, or wake episode was temporally compressed to unit length before the firing rates were averaged over multiple sequences and across neurons. Shading, ±s.e.m. Inset, the firing rates during the NREM periods preceding (NR_{pre}) and following REM (NR_{post}) were not significantly different (P = 0.51, $T(11)$ = 0.68, paired *t*-test). (**b**) Similar to (**a**), but for the NREM→Wake→NREM transition sequence. The mean firing rates of the REM-on neurons during NR_{pre} and NR_{post} were similar (P = 0.06, $T(11)$ = -2.11, paired *t*-test). (**c**) The firing rate of REM-on neurons during the inter-REM interval following a short (≤ 90 s) REM episode were similar to that following a long (> 90 s) REM episode (P = 0.18, $T(6)$ = -1.51, paired *t*-test). (**d**) The firing rates of REM-on neurons during the subsequent inter-REM interval showed no significant correlation with REM episode duration. Each dot represents the activity of a unit during a single inter-REM interval (n = 56). Line, linear fit (R = 0.07, P = 0.60, $T(54)$ = 0.52, linear regression). (**e**) The duration of an inter-REM interval was not significantly correlated with the firing rate of REM-on neurons during the interval. Each dot represents the activity of a unit during a single inter-REM interval (n = 56). Line, linear fit (R = 0.05, P = 0.70, $T(54)$ = 0.39).

References

1 Weber, F. *et al.* Control of REM sleep by ventral medulla GABAergic neurons. *Nature* **526**, 435-438 (2015).
