# Supplementary material for: Regulation of REM and Non-REM Sleep by Periaqueductal GABAergic Neurons
Source: Nat Commun. 2018 Jan 24;9:354. doi: 10.1038/s41467-017-02765-w (PMC5783937; doi:10.1038/s41467-017-02765-w)
Supplement: Supplementary file 3 — Description of Additional Supplementary Files [file 41467_2017_2765_MOESM3_ESM.pdf]

Description of Additional Supplementary Files

File Name: Supplementary Movie 1
Description: **Firing rates of an identified vlPAG GABAergic neuron recorded during sleep.** The video shows an example optrode recording of a vlPAG GABAergic unit. EEG spectrogram, EMG amplitude, color-coded brain states, and firing rates are shown on the right. The time points of single spikes are represented as vertical lines on the bottom left. 10x speedup.

File Name: Supplementary Movie 2
Description: **Sleep-wake activity of vlPAG GABAergic neurons measured with calcium imaging.** The video shows an example imaging session of vlPAG GABAergic neurons expressing GCaMP6 (left). EEG spectrogram, EMG amplitude, and $\Delta F/F$ traces are shown on the right. The ROIs corresponding to the $\Delta F/F$ traces are indicated by arrows in the imaging video. Scale bar, 100 µm; 10x speedup.
